# Supplementary material for: Long Period Grating Mach–Zehnder Interferometer Based Immunosensor with Temperature and Bulk Refractive Index Compensation
Source: Biosensors (Basel). 2022 Nov 30;12(12):1099. doi: 10.3390/bios12121099 (PMC9775309; doi:10.3390/bios12121099)
Supplement: Supplementary file 1 [file biosensors-12-01099-s001.zip › biosensors-1947394-supplementary.pdf]

Long Period Grating Mach–Zehnder Interferometer Based Immunosensor with Temperature and Bulk Refractive Index Compensation

Peizhou Wu, Liangliang Liu, Stephen P. Morgan , Ricardo Correia and Serhiy Korposh *

Optics and Photonics Research Group, University of Nottingham, University Park, Nottingham NG7 2RD, UK

I. Derivation of the X-Axis of Figure 3b in Manuscript

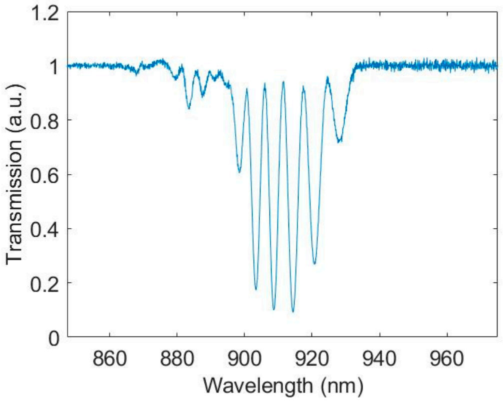

Figure S1a. The transmission spectrum of LPGMZI

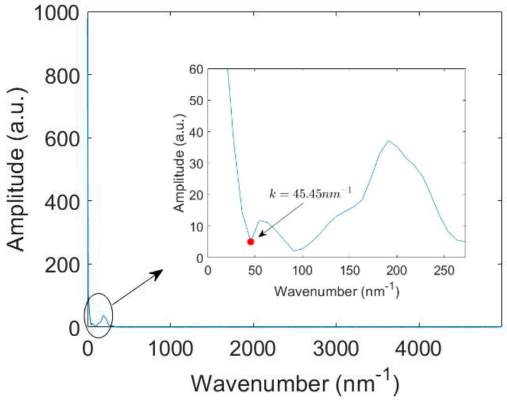

Figure S1b. The frequency-domain signal of LPGMZI's transmission spectrum

The Discrete Fourier Transform (DFT) and inverse DFT of a signal *x*[n] are shown in Equation(1a) and Equation(1b) respectively:

$$X[k] = \sum_{n=0}^{N-1} x[n] e^{-\frac{j2\pi nk}{N}} \quad (1a)$$

$$x[n] = \frac{1}{N} \sum_{k=0}^{N-1} X[k] e^{j\frac{2\pi nk}{N}} \quad (1b)$$

To get the frequency-domain representation of the transmission spectrum of LPGMZI, apply Equation(1a) to the signal in Figure(1a). As the resolution of spectrometer is 0.11nm, *n*=847.31nm, 847.31nm+1×0.11*nm*, 847.31nm+2×0.11*nm*, ⋯, 847.31nm+(*N*-1)×0.11*nm*. *N* is the number of sample points on the spectrum. To make *n*×*k* dimensionless, *k*=0, $\frac{1}{0.11nm}$, 2×$\frac{1}{0.11nm}$, ⋯, (*N*-1)×$\frac{1}{0.11nm}$. *k*=0, 9.09nm^{-1}, 18.18nm^{-1}, ⋯, (*N*-1) ×nm^{-1}. The unit of *k* is wavenumber (nm^{-1}).

II. Non-specific Binding of IgM to Bare LPG

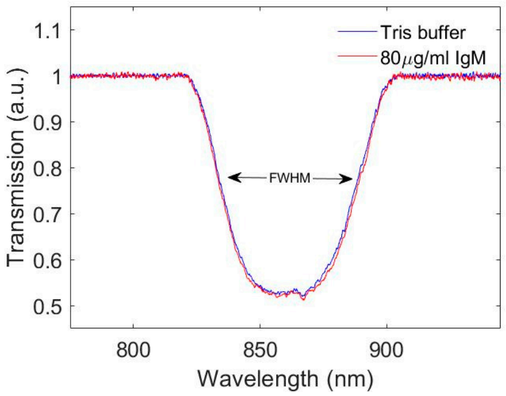

Figure S2a. The transmission spectra of single LPG working around PMTP

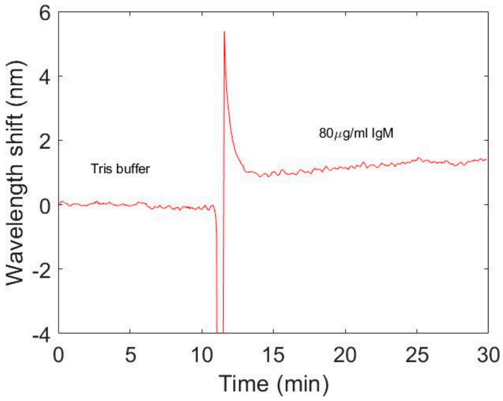

Figure S2b. The real-time data of the IgM measurement

The single LPG works around Phase Matching Turning Point (PMTP). As the IgM concentration increases, the U-shape attenuation of LPG goes deeper (Figure 2a). It is because of the non-specific binding of IgM molecules to bare LPG's surface. The Full Width Half Maximum (FWHM) is monitored during the measurement (Figure 2b). The wavelength shift is nearly 1.8nm at 80μg/ml IgM concentration.

III. The IgM Measurement with the Whole LPGMZI Immersed

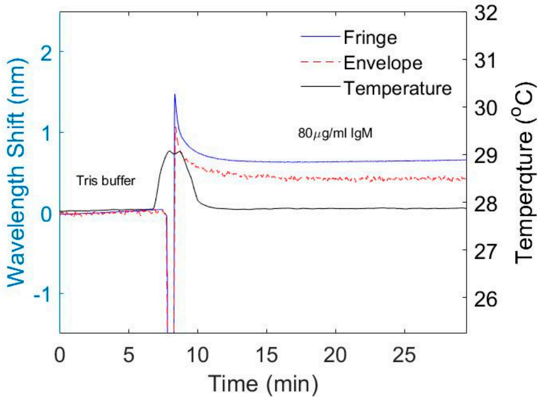

Figure S3. The IgM measurement with the whole LPGMZI immersed.

The whole LPGMZI (two LPGs and the connecting section) is immersed into solution during the measurement. As shown in Figure 3, both the fringe and envelope shift when the LPGMZI is immersed into 80μg/ml IgM solution. The temperature is measured with a thermocoupler (PICO Technology TC-08). Temperature remains constant. The wavelength shift of envelope is caused by the non-specific binding of IgM to bare LPGs' surfaces (Not bulk RI). The connecting section is functionalized with anti-IgM. That's why the fringe's wavelength shift is greater than the envelope's wavelength shift.

IV. The Measurement of Different Buffer Solutions

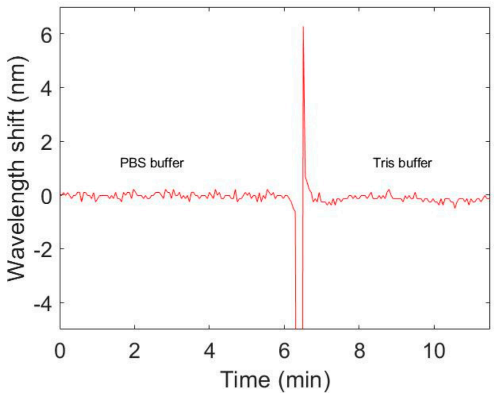

Figure S4. The measurement of PBS buffer and Tris buffer with single LPG.

As shown in Figure 4. Change from PBS buffer to Tris buffer won't affect LPG too much. The wavelength almost remains constant.

V. Repeatability and Stability Test of LPGMZI

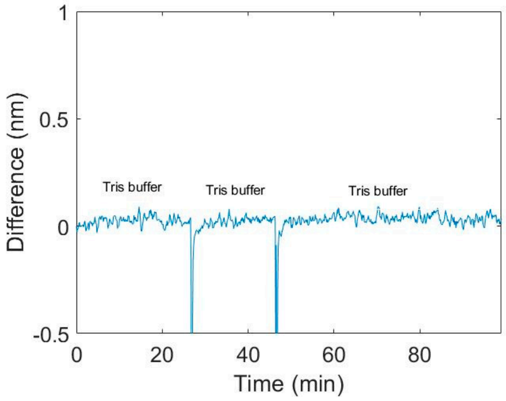

Figure S5. The test on LPGMZI by immersing the connecting section into Tris buffer solution for three times

The connecting section of LPGMZI is immersed into Tris buffer solution for three times. The difference between the wavelengths of fringe and envelope is calculated. It can be told that the baseline is stable, which is evidence of good repeatability and stability. The standard deviation of the differences on the three steps is 0.0055nm.
